# Identification and Characterization of Genes Related to Resistance of *Autographa californica* Nucleopolyhedrovirus Infection in *Bombyx mori*

**DOI:** 10.3390/insects13050435

**Published:** 2022-05-06

**Authors:** Yunhui Kong, Lingling Sun, Yaling Tang, Jiashuang Li, Sheng Qin, Muwang Li

**Affiliations:** 1 Jiangsu Key Laboratory of Sericultural Biology and Biotechnology, School of Biotechnology, Jiangsu University of Science and Technology, Zhenjiang 212100, China; kongyunhui1@gmail.com (Y.K.); sunlingling0828@163.com (L.S.); tangyaling714@163.com (Y.T.); ljs990302@163.com (J.L.); 2 Key Laboratory of Silkworm and Mulberry Genetic Improvement, Ministry of Agriculture and Rural Affairs, Sericultural Research Institute, Chinese Academy of Agricultural Science, Zhenjiang 212100, China

**Keywords:** *Bombyx mori*, GWAS, NPC-1, AcMNPV

## Abstract

**Simple Summary:**

*Autographa californica* nucleopolyhedrovirus (AcMNPV) is a kind of baculovirus that was initially found and named for its host, but the previous study reveals several silkworm strains are preferentially susceptible to AcMNPV through intrahemocelical injection method. In the following study, genetics analysis showed that a set of potential genes which controlled resistance of AcMNPV was located on chromosome 3. In the present research, we performed Genome-Wide Association Studies to identify the gene that controls the resistance of AcMNPV, results show that the *Niemann-Pick C1 (NPC-1)* gene is strongly associated with this resistance. Then we found that there are several amino acid mutations in the protein sequence of *BmNPC1* between two different resistance strains of *Bombyx mori*. RNAi results showed that *BmNPC1* successfully suppressed virus infection ability and changed the expression pattern of viral genes.

**Abstract:**

In *Bombyx mori*, as an important economic insect, it was first found that some strains were completely refractory to infection with *Autographa californica* nucleopolyhedrovirus (AcMNPV) through intrahemocelical injection; whereas almost all natural strains had difficulty resisting *Bombyx mori* nucleopolyhedrovirus (BmNPV), which is also a member of the family Baculoviridae. Previous genetics analysis research found that this trait was controlled by a potentially corresponding locus on chromosome 3, but the specific gene and mechanism was still unknown. With the help of the massive silkworm strain re-sequencing dataset, we performed the Genome-Wide Association Studies (GWAS) to identify the gene related to the resistance of AcMNPV in this study. The GWAS results showed that the Niemann-Pick type C1 (NPC-1) gene was the most associated with the trait. The knockdown experiments in BmN cells showed that *BmNPC1* has a successful virus suppression infection ability. We found a small number of amino acid mutations among different resistant silkworms, which indicates that these mutations contributed to the resistance of AcMNPV. Furthermore, inhibition of the *BmNPC1* gene also changed the viral gene expression of the AcMNPV, which is similar to the expression profile in the transcriptome data of p50 and C108 strains.

## 1. Introduction

Baculoviruses are a very diverse group of viruses with large rod-shaped envelopes and double-stranded, circular, supercoiled genomes, with sizes varying from about 80 kb to over 180 kb, that encode between 90 and 180 genes [1]. It was reported that baculoviruses have different tropisms among lepidopteran insects, but each kind of baculovirus usually has its own narrow host range [2], determined by its host cell-specific factor (*hcf*) [3] and host range factor (*hrf*) [4]. *Autographa californica* nucleopolyhedrovirus (AcMNPV) is a kind of baculovirus—initially found in and named after its host, *Autographa californica*—could infect and spread at a wide range of lepidopteran insect larvae [5]. Another kind of baculovirus, *Bombyx mori* nucleopolyhedrovirus (BmNPV), is the most destructive virus in the sericulture industry. To date, the intrinsic infection mechanism of BmNPV in silkworms is indistinct; however, from the aspect of genomics, BmNPV shared an overall 90% identity at the amino acid sequence level and 38 homologous genes with AcMNPV. For this reason, understanding the specific mechanism of anti-AcMNPV may provide some new strategy for us to confront BmNPV [1].

Silkworm, *Bombyx mori,* an important economic insect in sericulture and was first reported as not the permissive host of AcMNPV [6,7]. However, in the following research, Guo et al. found that there are several silkworm strains which are preferentially susceptible to AcMNPV through the intrahemocelical injection method [8]. It was also found that the gene *sav* which controls susceptibility of silkworms to AcMNPV was following Mendel’s law of segregation and severed as a dominant inhibitor in nonpermissive silkworms. Xu et al. have investigated the responses of 448 silkworm strains against recombinant AcMNPV inoculation. The authors found that most of the strains were completely refractory to infection by AcMNPV through a luciferase assay, the genetics analysis showed that the potential gene to control resistance of AcMNPV was located on chromosome 3, but the specific gene was still unknown [9].

*NPC-1* (Niemann-Pick type C1) gene encodes a ubiquitous endolysosomal membrane protein involved in intracellular cholesterol transport, its mutations would cause Niemann-Pick type C disease, a neurological disorder [10,11,12]. Previous research has shown that it was also severe as a virus intracellular receptor for filovirus entry, such as Ebola virus and Marburg virus; the mutation of domain C would result in complete resistance to virus infection [13,14,15,16]. A recent study showed that NPC-1 was also an essential host factor for baculovirus infection in insect cells, it may interact with the GP64 of BmNPV to facilitate the virus’ entry [17].

In our previous study, we identified AcMNPV resistance of several silkworm strains, but the specific major gene was still unclear [18]. Based on our previous large-scale re-sequencing data of multiple strains of silkworm, genome wide association analysis (GWAS) was performed to identify the loci most related to AcMNPV resistance in the current study, the results showed that there are several consistent extracellular amino acid mutations among different resistant silkworm strains [19]. The follow-up study showed that the inhibition of *BmNPC1* could decrease the propagation and alter the expression pattern of the AcMNPV.

## 2. Materials and Methods

### 2.1. Sample Preparation

All the silkworm strains were maintained in the Key Laboratory of Sericulture, Sericultural Research Institute, Chinese Academy of Agricultural Science. The larvae were fed with fresh mulberry leaves in conditions of 25 ± 1 °C, 75 ± 5% relative humidity, and a 12 h day/night cycle.

The resistance of strains was determined at fifth instars, based on our manual validation and previous studies [8,9,18,20], the detail phenotype information was shown in Table S1.

### 2.2. Genome-Wide Association and Population Genetic Analysis

Genome re-sequencing data of silkworms were obtained from previous research, which is available in NCBI Sequence Read Archive (SRA) database under the accession SRP119041 [19]. The raw reads were cleaned by fastp and mapped to reference genomes from KAIKObase (ver. 4.0.0) with bwa [21,22,23]. PCR duplications were removed by GATK MarkDuplicates, SNP calling and quality control followed the GATK best practices with default parameters [24,25]. The SNP was filtered by minor allele frequencies (MAF > 0.05) and missing rates (GENO > 0.05) with plink 1.9 [26]. R package rMVP was used to perform a generalized linear model (GLM) with kinship matrix as a covariate, the significant threshold was determined based on the Bonferroni adjustment method: threshold = 0.01/Total number of SNP) [27]. Linkage disequilibrium analysis was performed by PopLDdecay [28]. The transmembrane domain of BmNPC1 was predicted by TMHMM 2.0 web server.

### 2.3. Virus Gene Expression Profile Analysis based on RNA-Sequencing Data

In our previous study, we have studied the host gene expression infected by AcMNPV via RNA-seq between the p50 strain and C108 strains while the virus gene expression pattern was not considered. Thus, we reanalyzed the RNA-seq data from the aspects of the virus. Briefly, the raw reads were cleaned by fastp and mapped to AcMNPV reference genomes from the NCBI RefSeq genome (GCF_000838485.1) with Hisat2 [29]. FeatureCounts was used to obtain the raw counts of every virus gene [30]. For variant calling, STAR twopassMode was used following the GTAK RNA-seq short variant discovery workflow [31]. The RNA-seq data was available at NCBI Sequence Read Archive (SRA) database under the accession SRR15247045-SRR15247052 [18].

### 2.4. Genome and RNA Extraction

Genomic DNA of cells was independently extracted by Genomic DNA Extraction Kit according to the manufacturer’s instructions (Sangon, Shang Hai, China). Total RNAs of BmN cell and multi silkworm strain were extracted using TRIzol reagent (Invitrogen, Waltham, USA) according to the manufacturer’s instructions, then precipitated and purified with isopropyl alcohol and dissolved in DEPC water. The assessing optical density (OD) absorbance ratio of 260/280 was determined. The concentration of RNA was detected using a NanoDrop 2000 spectrophotometer. RNA integrity was checked by 1% agarose gel electrophoresis. A total of 1.0 µg of RNA was reverse-transcribed in vitro by the PrimeScriptTM RT reagent kit according to the manufacturer’s instructions.

### 2.5. PCR and qRT-PCR

Full-length CDS of *BmNPC1* gene in of BmN cell and multi silkworm strain was cloned following the previous study [32]. Then PCR products were inserted into the PMD19-T vector (TaKaRa, Dalian, China) according to the manufacturer’s instructions, then sequenced at the Sangon Biotech (Shanghai, China). All nucleotide sequences were translated into protein sequences and then aligned by Clustal Omega [33]. Gene structure visualization was performed by R package ggbio and ggplot2 [34]. All the variants were checked manually in the IGV browser to ensure the consistency of the genotype with our previous re-sequencing data [19,35].

qRT-PCR was used to detect the gene expression levels and copy numbers of AcMNPV. The specific primers used in qRT-PCR were designed by the NCBI Primer-BLAST software (https://www.ncbi.nlm.nih.gov/tools/primer-blast/) (accessed on 7 September 2021) and are shown in Table S2. The reaction mixtures were prepared using the NovoStart^®^SYBR qRT-PCR SuperMix Plus kit (Novoprotein Technology Ltd., Nanjing, China) according to the manufacturer’s instructions. Briefly, a 10 µL qRT-PCR reaction system was used, including 5 µL of 2× NovoStart^®^SYBR qRT-PCR SuperMix Plus, 0.5 µL of upstream and downstream primers, 1 µL of the template, and 3.0 µL of ddH_2_O. The reactions were performed on the LightCycler^®^ 96 System (Roche, Basel, Switzerland). The following qRT-PCR protocol was used: one cycle at 95 °C for 5 min, followed by 40 cycles at 95 °C for 20 s, and 56 °C for 60 s. The 2−∆∆CT method was adopted to calculate the relative expression level. Each group was repeated three times. *Bombyx mori* glyceraldehyde-3-phosphate dehydrogenase (*BmGAPDH*) was used as the reference gene. The *late expression factor 3* (*lef3*) was used to detect the relative copy numbers of AcMNPV, all the primes were listed in Table S2.

### 2.6. Synthesis of siRNA

Two targets for functional domains of *BmNPC1* were designed by Thermo Fisher Scientific BLOCK-iT™ RNAi Designer (https://rnaidesigner.thermofisher.com/rnaiexpress) (accessed on 17 October 2021) to knockdown *BmNPC1*. The siRNA Oligos were synthesized by SUNYA Biotechnology (Zhejiang, China) and are listed in Table 1. The siRNAs were synthesized by the In Vitro Transcription T7 Kit (TaKaRa Biotechnology Co. Ltd., Dalian, China) according to the manufacturer’s instructions.

BmNPC1-1 Oligo (siNPC1) synthetic siRNA was used to inhibit BmNPC1 expression in BmN cells, and RFP-Oligo synthetic siRNA was used as a control. The kit uses T7 RNA polymerase to transcribe DNA sequences downstream of the promoter into highly synthetic single-stranded RNA using linear double-stranded DNA containing the T7 promoter sequence as a template. Absorbance ratio of 260/280 and concentration. The quality of the synthesized siRNA was checked by 3% agarose gel electrophoresis at 140 V for 10 min. Qualified siRNAs was stored at −80 °C until use.

### 2.7. BmN Cell Culture and Transfection

The BmN cell line was obtained from the silkworm ovary. It was cultured in TC100 medium pH 6.2 with 10% fetal bovine serum (FBS) and 1% penicillin-streptomycin solution at 28 °C. The culture medium was replaced every 4 days.

The siRNA was transfected by NeofectTM DNA transfection reagent (NEOFECT, Beijing, China) according to the manufacturer’s instructions. The BmN cells were cultured in the 60 mm dish. Briefly, each dish required 4.0 µg siRNA. The siRNA was mixed with 200 µL TC100 without FBS and 4.0 µL Neofect transfection reagent.

### 2.8. Statistical Analysis

The statistical differences among three biological duplicates were determined with ANOVA and Student’s *t*-test by R. The level of statistical significance was set at *, *p* < 0.05; **, *p* < 0.01; and ***, *p* < 0.001.

## 3. Results

### 3.1. Genome-Wide Association Analysis

**Figure 1:**
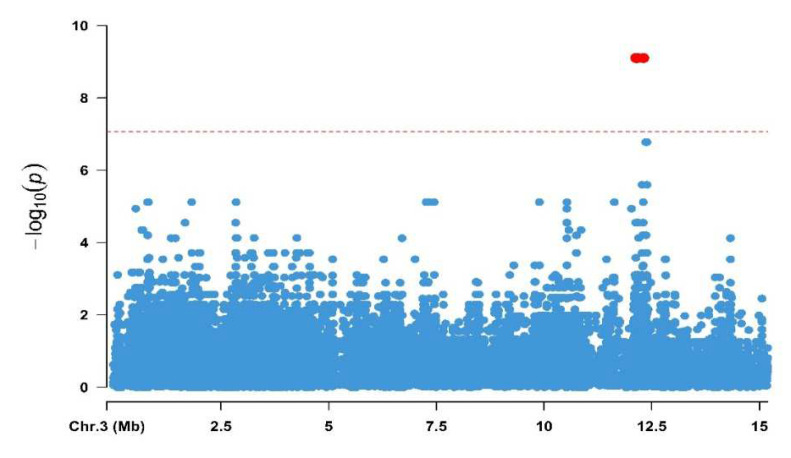
Manhattan plots of −log_10_ transformed observed *p*-values shown that only two loci were strong associated with resistance of AcMNPV.

Since the genetic analysis from the previous study showed that the gene for controlling the resistance of AcMNPV was located on chromosome 3, only the variants located on chromosome 3 were called [9]. GWAS analysis also manifested decent power, the Manhattan plot showed that there were only two peaks which were strongly associated with the trait (Figure 1).

The detailed peak information is listed in Table 2, two significant outlier peaks of the nearest genes were characterized, and it was also shown that linkage disequilibrium decays rapidly, thus we considered both genes were associated with AcMNPV resistance (Figure S1). Among these peaks, peak1 (12.13 Mb–12.16 Mb) is located in the intron of chloride intracellular channel gene while peak2 (12.30 Mb) is located in the 3′UTR and CDS of *Niemann-Pick C1* (*NPC-1*) gene. We performed RNAi to the *chloride intracellular channel* gene and found that the copy numbers of the virus were not significantly changed. As previous report that the *NPC-1* gene was an essential host factor in BmNPV infection [17], therefore the *NPC-1* was selected as a candidate gene for further research.

### 3.2. Several Consistent Amino Acid Mutations among Different Resistance Silkworm Strains

**Figure 2:**
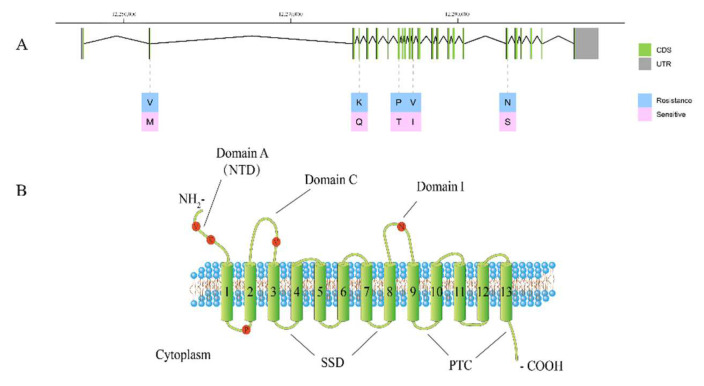
(**A**) Gene structure of BmNPC1 and missense mutation loci in different resistance silkworm group; (**B**) Protein structure of BmNPC1 and missense mutation loci in different domains.

The CDS region of *BmNPC1* was cloned and sequenced in different resistance silkworms, multi-sequence alignment shows that there are several consistent amino acid missense mutations among different resistance silkworms, which means that there are at least two distinct haplotypes of *BmNPC1* in the silkworm group (Figure 2A, File S1). The prediction of transmembrane structure results shows that four out of five amino acid mutations are extracellular (Figure 2B). These mutations may contribute to the resistance of AcMNPV. For example, we observed isoleucine to valine mutation in domain C of *BmNPC1*, which was considered an interaction region with BmNPV. The expression of BmNPC1 was also compared betweenp50 and C108 strains. The qRT-PCR result showed that the expression level of *BmNPC1* was one times higher in p50 strain (Figure S2). It implied that *BmNPC1* had lower expression in resistance strain.

### 3.3. Inhibition of BmNPC1 Decreases Propagation of the AcMNPV

**Figure 3:**
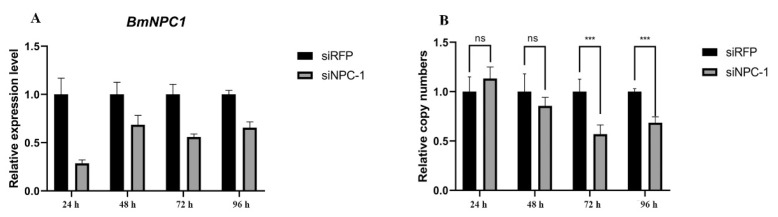
(**A**) The relative expression levels of *BmNPC1* in BmN cells after RNAi; (**B**) The relative copy numbers of virus between RFP and RNAi. The level of statistical significance was set at ns, *p*﹥0.05 and ***, *p* < 0.001.

Although the AcMNPV genome has a high consistency with BmNPV, AcMNPV can only infect a part of strains via intrahemocelical injection. Whether *BmNPC1* play an important role in AcMNPV infection is still unclear. The siRNA was synthesized and transfected into BmN cells to investigate whether *BmNPC1* has the ability to repress virus entry cells or propagation. The virus was added at 24 h after siRNA transfection. As our previous study performed, 24 h was selected as the time point for further analysis [18]. The qRT-PCR results showed that the expression of *BmNPC1* was successfully dropped by 70% at 24 h, and maintained a 50% decrease over the following time (Figure 3A). Copy numbers of the virus decreased by 56.9% at 72 h and by 68.6% at 96 h compared with the control group, respectively. It indicated that *BmNPC1* also played an essential function during AcMNPV infection (Figure 3B).

**Figure 4:**
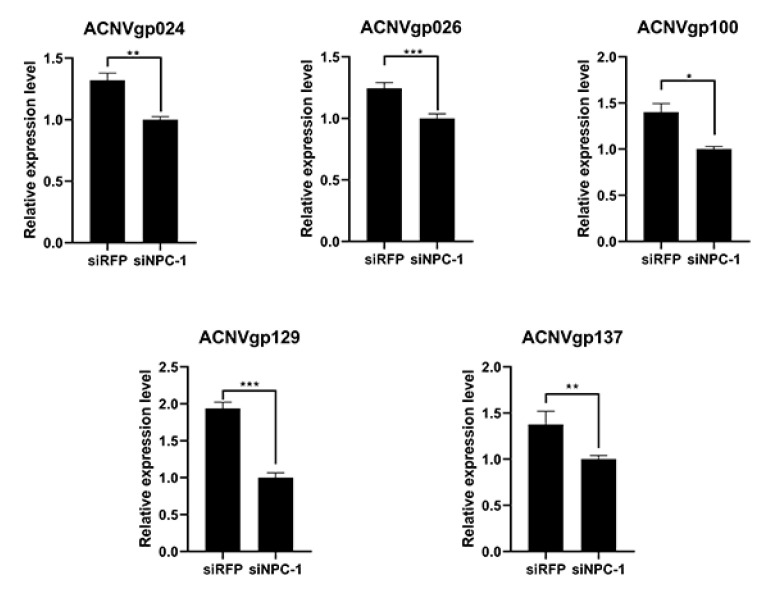
Relative expression levels of virus genes in BmN cells after *BmNPC1* RNAi 72 h. The level of statistical significance was set at *, *p* < 0.05; **, *p* < 0.01; and ***, *p* < 0.001.

To test whether inhibition of *BmNPC1* can lead to a change in the expression of AcMNPV genes, the virus gene expression at 72 h was also determined after knockdown of *BmNPC1*. The results showed that the expression levels of most viral genes had changed, but not by the same amount, such as the *gp64* (ACNVgp100) gene which was decreased to 50% of the control while the others were decreased only to 30% (Figure 4).

### 3.4. Distinct Virus Gene Expression Pattern between C108 and p50 Strain

AcMNPV cannot infect *B. mori* via oral infection, but it can infect a parts strains via intrahemocelical injection. In our previous study, we found that the response of intrahemocelical injection between C108 (resistant strain) and p50 (susceptible strain) strain was different, but the virus expression pattern was not characterized [18]. The transcriptome data (Table S3) showed that 68.59% of genes of AcMNPV (107 out of 156) were differentially expressed between C108 and p50. Compared with p50, 58 genes of AcMNPV were downregulated and 49 genes of AcMNPV were upregulated in C108. As a budded virus (BV) fusion protein, GP64 plays an important role in AcMNPV invention. The expression level of *gp64* (ACNVgp129) in p50 was 3.02-fold higher than that in C108. The expression of *fp* (ACNVgp062) and fibrous body protein (ACNVgp138) was extremely inhibited in C108.

**Figure 5:**
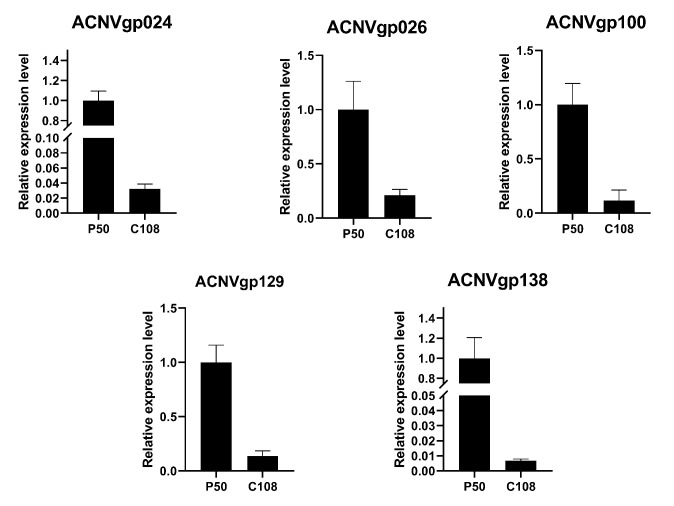
Relative expression levels of virus genes in p50 and C108 strain.

We observed that a part of late expression genes was significantly suppressed in C108. Protein kinase interacting protein (PKIP, ACNVgp024) which interacts with AcMNPV protein kinase I (PK-1, ACNVgp010) plays an important role in nucleocapsid assembly [36]. The expression level of *PKIP* in p50 was 4.82-fold in C108. *Bm17* (ACNVgp026), a late gene, might control the spread speed of AcMNPV in BmN cells. The expression level of *Bm17* in p50 was 1.9-fold than that in C108. *Lef-5* (Late expression factor 5 (ACNVgp100) which is required for late transcription activity and infection was also inhibited in C108. Its expression level in C108 dropped to 15.71% of that in p50. It indicated that the regulation of AcMNPV life cycle was seriously affected in the C108 strain. The expression of these genes has been further verified by qRT-PCR (Figure 5).

## 4. Discussion

BmNPV is one of the main viral pathogens to *Bombyx mori*, which causes tremendous economic loss every year, but there still is limited knowledge for virus invasion process and immune evasion mechanism [37,38]. Recent studies reveal that there are several invasion mechanisms of BmNPV, such as cholesterol mediated macropinocytic endocytosis and clathrin medicated endocytosis [37,38,39,40]. The glycoprotein *gp64* gene of baculovirus and its homolog has been proven its crucial role in the viral host determination. There are only 22 different amino acids between the GP64 protein of AcMNPV and BmNPV, while they have a distinct natural host [41]. Thus, the AcMNPV is a valuable model for researching the resistant mechanism to BmNPV in *Bombyx mori*. A previous study shows that the cholesterol recognition amino acid consensus (CRAC) domain of GP64 serves as an anchor in the AcMNPV entry and is essential for the efficiency of infection [42]. A recent study showed that the CRAC of GP64 of BmNPV also interacted with the cholesterol in the cell plasma membrane [43].

The *NPC-1* is a multi-functional gene which not only serves as an intercellular cholesterol transport protein, but also as a cytomembrane receptor to mediate cell infection by a wide range of viruses [17,44]. To date, multiple virus families have demonstrated that their penetration process needs the participation of the NPC-1 protein while having different features of the molecular mechanism [17]. The NPC-1 protein mainly has three extracellular domains (domain A, domain I, and domain C). All of them have reported contributions to virus resistance, especially domain C. Domain C has been proven as critical for virus entry and directly interacts with the glycoprotein of the Ebola virus and BmNPV [15,45,46]. A recent study shows that a few mutations of amino acids in domain C could result in differential susceptibility to filoviruses, which indicates that the interaction between NPC-1 protein and virus glycoprotein can be affected by a few amino acid residues [44]. Co-immunoprecipitation experiments in silkworm show that domain C of NPC-1 protein also interacts with GP64 of BmNPV, which means that AcMNPV may similarly invade cells via its highly conserved GP64 protein [17]. In the current study, we identified isoleucine to valine mutation located in domain C, two mutations located in N-terminal (domain) which is also reported as a cholesterol binding pocket, and one mutation in domain A [46,47]. RNAi experiment showed that inhibition of *BmNPC1* can prevent the invasion of AcMNPV. This implied that *BmNPC1* also had a great contribution to AcMNPV infection

In addition, the absence of *BmNPC1* also could change the expression level of virus genes. We observed that a part of late expression genes were significantly inhibited in C108—for example, *gp64* and *fp*. This will affect the late assembly of AcMNPV. The lack of GP64 results in viruses that replicate in a single cell, but cannot bud out and infect surrounding cells [48,49]. Expression changes of *gp64* and *fp* might be because when baculovirus enters the cell via a clathrin–dynamin-independent pathway, the genetic material of the virus is hard to enter the nucleus of host cells [42]. In our work, a reduction in *gp64* expression was also observed when *BmNPC1* was inhibited in BmN cells. Our finding suggested that *BmNPC1* participated in AcMNPV infection, and its mutation provided the AcMNPV resistance in the C108 strain.

## Figures and Tables

**Table 1:** List of primer sequences used to synthesize siRNA.

Primer Names	Sequences (5′–3′)
BmNPC1-1 Oligo-1	GATCACTAATACGACTCACTATAGGGCGTGCTGCAATTACGAACAACTGAATT
BmNPC1-1 Oligo-2	AATTCAGTTGTTCGTAATTGCAGCACGCCCTATAGTGAGTCGTATTAGTGATC
BmNPC1-1 Oligo-3	AACGTGCTGCAATTACGAACAACTGAACCCTATAGTGAGTCGTATTAGTGATC
BmNPC1-1 Oligo-4	GATCACTAATACGACTCACTATAGGGTTCAGTTGTTCGTAATTGCAGCACGTT
BmNPC1-2 Oligo-1	GATCACTAATACGACTCACTATAGGGGAGCAAATACTTGAAGCCAGTTCAATT
BmNPC1-2 Oligo-2	AATTGAACTGGCTTCAAGTATTTGCTCCCCTATAGTGAGTCGTATTAGTGATC
BmNPC1-2 Oligo-3	AAGAGCAAATACTTGAAGCCAGTTCAACCCTATAGTGAGTCGTATTAGTGATC
BmNPC1-2 Oligo-4	GATCACTAATACGACTCACTATAGGGTTGAACTGGCTTCAAGTATTTGCTCTT
RFP-Oligo-1	GATCACTAATACGACTCACTATAGGGGCACCCAGACCATGAGAATTT
RFP-Oligo-2	AAATTCTCATGGTCTGGGTGCCCCTATAGTGAGTCGTATTAGTGATC
RFP-Oligo-3	AAGCACCCAGACCATGAGAATCCCTATAGTGAGTCGTATTAGTGATC
RFP-Oligo-4	GATCACTAATACGACTCACTATAGGGATTCTCATGGTCTGGGTGCTT

**Table 2:** GWAS peak information.

Chr	Position	Ref	Alt	*p*-Value	Nearest Gene
3	12,131,761	C	T	7.88 × 10^−10^	Chloride intracellular channel
3	12,131,766	A	T	7.88 × 10^−10^	Chloride intracellular channel
3	12,156,953	A	C	7.88 × 10^−10^	Chloride intracellular channel
3	12,166,583	T	C	7.88 × 10^−10^	Chloride intracellular channel
3	12,167,055	T	G	7.88 × 10^−10^	Chloride intracellular channel
3	12,306,266	A	C	7.88 × 10^−10^	Niemann-Pick C1
3	12,306,312	G	A	7.88 × 10^−10^	Niemann-Pick C1
3	12,306,315	G	A	7.88 × 10^−10^	Niemann-Pick C1

## Data Availability

The data presented in this study are available in supplementary material.

## Supplementary Materials

The following supporting information can be downloaded at: https://www.mdpi.com/article/10.3390/insects13050435/s1, Figure S1: Linkage disequilibrium levels of silkworm population; Figure S2: The relative expression leveFls of *BmNPC1* in hemolymph of p50 and C108 strain on the first day of the fifth instar. Table S1: Phenotype information and SRA accession of silkworm strains; Table S2: Specific primers used in qRT-PCR; Table S3: Expression pattern of AcMNPV in C108 and p50 stain; File S1: Multiple sequence alignment of BmNPC1 protein among different resistance silkworm.
