# Supplementary material for: Identification and Characterization of Genes Related to Resistance of Autographa californica Nucleopolyhedrovirus Infection in Bombyx mori
Source: Insects. 2022 May 6;13(5):435. doi: 10.3390/insects13050435 (PMC9144136; doi:10.3390/insects13050435)
Supplement: Supplementary file 1 [file insects-13-00435-s001.zip › File S1.pdf]

CLUSTAL O(1.2.4) multiple sequence alignment

```
p50             MADRGSFLRRFLSALFFLLVLVEHIPSALAEDGHCIWYGVCANSTEHKMMNCFYDGPAKP 60
Nistari         MADRGSFLRRFLSALFFLLVLVEHIPSALAEDGHCIWYGVCANSTEHKMMNCFYDGPAKP 60
Cell            MADRGSFLRRFLSALFFLLVLVEHIPSALAEDGHCIWYGVCANSTEHKMMNCFYDGPAKP 60
C108            MADRGSFLRRFLSALFFLLVLVEHIPSALAEDGHCIWYGVCANSTEHKMVNCFYDGPAKP 60
JingSong        MADRGSFLRRFLSALFFLLVLVEHIPSALAEDGHCIWYGVCANSTEHKMVNCFYDGPAKP 60
                *************************************************:**********

p50             IEEKAMKIIEQYCPEIAKGGYSCCNYEQLNQLKVNIALAENLLGRCPTCFQNFLKPICGM 120
Nistari         IEEKAMKIIEQYCPEIAKGGYSCCNYEQLNQLKVNIALAENLLGRCPTCFQNFLKPICGM 120
Cell            IEEKAMKIIEQYCPEIAKGGYSCCNYEQLNQLKVNIALAENLLGRCPTCFQNFLKPICGM 120
C108            IEEKAMKIIEQYCPEIAKGGYSCCNYEQLNQLKVNIALAENLLGRCPTCFQNFLKPICGM 120
JingSong        IEEKAMKIIEQYCPEIAKGGYSCCNYEQLNQLKVNIALAENLLGRCPTCFQNFLKPICGM 120
                ************************************************************

p50             ACAVDQSKYLKPVQTEQFNGTHRRISEIDYYLSKTFMDGIYNSCSEVKLTSTNERSIKVM 180
Nistari         ACAVDQSKYLKPVQTEQFNGTHRRISEIDYYLSKTFMDGIYNSCSEVKLTSTNERSIKVM 180
Cell            ACAVDQSKYLKPVQTEQFNGTHRRISEIDYYLSKTFMDGIYNSCSEVKLTSTNERSIKVM 180
C108            ACAVDQSKYLKPVKTEQFNGTHRRISEIDYYLSKTFMDGIYNSCSEVKLTSTNERSIKVM 180
JingSong        ACAVDQSKYLKPVKTEQFNGTHRRISEIDYYLSKTFMDGIYNSCSEVKLTSTNERSIKVM 180
                *************:**********************************************

p50             CGQWGDACSAQRWFDNMGDASNYQVPFQINYKATDVPVDGYTPYAPIAEPCNVGTKGMPG 240
Nistari         CGQWGDACSAQRWFDNMGDASNYQVPFQINYKATDVPVDGYTPYAPIAEPCNVGTKGMPG 240
Cell            CGQWGDACSAQRWFDNMGDASNYQVPFQINYKATDVPVDGYTPYAPIAEPCNVGTKGMPG 240
C108            CGQWGDACSAQRWFDNMGDASNYQVPFQINYKATDVPVDGYTPYAPIAEPCNVGTKGMPG 240
JingSong        CGQWGDACSAQRWFDNMGDASNYQVPFQINYKATDVPVDGYTPYAPIAEPCNVGTKGMPG 240
                ************************************************************

p50             CSCLDCEASCPAPPPRPPPPQPFSIAGFDGYAIVMTIVFCIFSTLFLTGVFCCNQTENLV 300
Nistari         CSCLDCEASCPAPPPRPPPPQPFSIAGFDGYAIVMTIVFCIFSTLFLTGVFCCNQTENLV 300
Cell            CSCLDCEASCPAPPPRPPPPQPFSIAGFDGYAIVMTIVFCIFSTLFLTGVFCCNQTENLV 300
C108            CSCLDCEASCPAPPPRPPPPQPFSIAGFDGYAIVMTIVFCIFSTLFLTGVFCCNQTENLV 300
JingSong        CSCLDCEASCPAPPPRPPPPQPFSIAGFDGYAIVMTIVFCIFSTLFLTGVFCCNQTENLV 300
                ************************************************************

p50             ECGPETSPLHERRASTYPPLLHTHGVSSDNNQEMGTNASGTRWTMEATDGDETDATFLEK 360
Nistari         ECGPETSPLHERRASTYPPLLHTHGVSSDNNQEMGTNASGTRWTMEATDGDETDATFLEK 360
Cell            ECGPETSPLHERRASTYPPLLHTHGVSSDNNQEMGTNASGTRWTMEATDGDETDATFLEK 360
C108            ECGPETSPLHERRASTYPPLLHTHGVSSDNNQEMGTNASGTRWTMEAPDGDETDATFLEK 360
JingSong        ECGPETSPLHERRASTYPPLLHTHGVSSDNNQEMGTNASGTRWTMEAPDGDETDATFLEK 360
                *********************************************** ************

p50             LGAATESKIEDFFQWWGCVMASSPWLVLFSGLCVVVILGSGITYMQVTTDPVELWASPTS 420
Nistari         LGAATESKIEDFFQWWGCVMASSPWLVLFSGLCVVVILGSGITYMQVTTDPVELWASPTS 420
Cell            LGAATESKIEDFFQWWGCVMASSPWLVLFSGLCVVVILGSGITYMQVTTDPVELWASPTS 420
C108            LGAATESKIEDFFQWWGCVMASSPWLVLFSGLCVVVILGSGITYMQVTTDPVELWASPTS 420
JingSong        LGAATESKIEDFFQWWGCVMASSPWLVLFSGLCVVVILGSGITYMQVTTDPVELWASPTS 420
                ************************************************************

p50             RSRVERQRFDSYFEPFYRTEMLIISSKGLPEIEHANLTFGPVFNATFMLDVFDLQSKILE 480
```

```
Nistari      RSRVERQRFDSYFEPFYRTEMLIISSKGLPEIEHANLTFGPVFNATFMLDVFDLQSKILE 480
Cell         RSRVERQRFDSYFEPFYRTEMLIISSKGLPEIEHANLTFGPVFNATFMLDVFDLQSKILE 480
C108         RSRVERQRFDSYFEPFYRTEMLIISSKGLPEIEHANLTFGPVFNATFMLDVFDLQSKILE 480
JingSong     RSRVERQRFDSYFEPFYRTEMLIISSKGLPEIEHANLTFGPVFNATFMLDVFDLQSKILE 480
             ************************************************************
p50          LGNESGIQNICYAPLSSPFRGPVTSKDCVLQSVWGWWQNDKSEFSYEDNEHLDKILQCSS 540
Nistari      LGNESGIQNICYAPLSSPFRGPVTSKDCVLQSVWGWWQNDKSEFSYEDNEHLDKILQCSS 540
Cell         LGNESGIQNICYAPLSSPFRGPVTSKDCVLQSVWGWWQNDKSEFSYEDNEHLDKILQCSS 540
C108         LGNESGIQNICYAPLSSPFRGPVTSKDCVLQSVWGWWQNDKSEFSYEDNEHLDKILQCSS 540
JingSong     LGNESGIQNICYAPLSSPFRGPVTSKDCVLQSVWGWWQNDKSEFSYEDNEHLDKILQCSS 540
             ************************************************************
p50          NPISVDCLSSYGGPVLPGVALGGFLPRGDQLSAHAPYHRAQALILTFLVNNKQDKSQLKQ 600
Nistari      NPISVDCLSSYGGPVLPGVALGGFLPRGDQLSAHAPYHRAQALILTFLVNNKQDKSQLKQ 600
Cell         NPISVDCLSSYGGPVLPGVALGGFLPRGDQLSAHAPYHRAQALILTFLVNNKQDKSQLKQ 600
C108         NPISVDCLSSYGGPVLPGVALGGFLPRGDQLSAHAPYHRAQALVLTFLVNNKQDKSQLKQ 600
JingSong     NPISVDCLSSYGGPVLPGVALGGFLPRGDQLSAHAPYHRAQALVLTFLVNNKQDKSQLKQ 600
             *******************************************:****************
p50          ALEWEKTFIAFMKNYTEKAMPGYMDIAYTSERSIEDELDRESKSDVYTILVSYFIMFAYI 660
Nistari      ALEWEKTFIAFMKNYTEKAMPGYMDIAYTSERSIEDELDRESKSDVYTILVSYFIMFAYI 660
Cell         ALEWEKTFIAFMKNYTEKAMPGYMDIAYTSERSIEDELDRESKSDVYTILVSYFIMFAYI 660
C108         ALEWEKTFIAFMKNYTEKAMPGYMDIAYTSERSIEDELDRESKSDVYTILVSYFIMFAYI 660
JingSong     ALEWEKTFIAFMKNYTEKAMPGYMDIAYTSERSIEDELDRESKSDVYTILVSYFIMFAYI 660
             ************************************************************
p50          AIALGRFTTFSRLLIDSKITLGLGGVVIVLASVVCSMGIFGFYGVAATLIIVEVIPFLVL 720
Nistari      AIALGRFTTFSRLLIDSKITLGLGGVVIVLASVVCSMGIFGFYGVAATLIIVEVIPFLVL 720
Cell         AIALGRFTTFSRLLIDSKITLGLGGVVIVLASVVCSMGIFGFYGVAATLIIVEVIPFLVL 720
C108         AIALGRFTTFSRLLIDSKITLGLGGVVIVLASVVCSMGIFGFYGVAATLIIVEVIPFLVL 720
JingSong     AIALGRFTTFSRLLIDSKITLGLGGVVIVLASVVCSMGIFGFYGVAATLIIVEVIPFLVL 720
             ************************************************************
p50          AVGVDNIFILVQTHQREPRRPDETVEQHIGRMLGKVGPSMFVTSVSESVCFFLGALSDMP 780
Nistari      AVGVDNIFILVQTHQREPRRPDETVEQHIGRMLGKVGPSMFVTSVSESVCFFLGALSDMP 780
Cell         AVGVDNIFILVQTHQREPRRPDETVEQHIGRMLGKVGPSMFVTSVSESVCFFLGALSDMP 780
C108         AVGVDNIFILVQTHQREPRRPDETVEQHIGRMLGKVGPSMFVTSVSESVCFFLGALSDMP 780
JingSong     AVGVDNIFILVQTHQREPRRPDETVEQHIGRMLGKVGPSMFVTSVSESVCFFLGALSDMP 780
             ************************************************************
p50          AVRAFALYAAVALLVDFLLQVTCFVALLALDTRRQLDNRFDIFCCLRGTKTDLAEQGEGA 840
Nistari      AVRAFALYAAVALLVDFLLQVTCFVALLALDTRRQLDNRFDIFCCLRGTKTDLAEQGEGA 840
Cell         AVRAFALYAAVALLVDFLLQVTCFVALLALDTRRQLDNRFDIFCCLRGTKTDLAEQGEGA 840
C108         AVRAFALYAAVALLVDFLLQVTCFVALLALDTRRQLDNRFDIFCCLRGTKTDLAEQGEGA 840
JingSong     AVRAFALYAAVALLVDFLLQVTCFVALLALDTRRQLDNRFDIFCCLRGTKTDLAEQGEGA 840
             ************************************************************
p50          LYNLFQHFYVPFLMKREVRASVMIIFFAWLCSSVAVAPHIDIGLDQELSMPHDSFQLKYF 900
Nistari      LYNLFQHFYVPFLMKREVRASVMIIFFAWLCSSVAVAPHIDIGLDQELSMPHDSFQLKYF 900
Cell         LYNLFQHFYVPFLMKREVRASVMIIFFAWLCSSVAVAPHIDIGLDQELSMPHDSFQLKYF 900
```

```
C108        LYNLFQHFYVPFLMKREVRASVMIIFFAWLCSSVAVAPHIDIGLDQELSMPHDSFQLKYF 900
JingSong    LYNLFQHFYVPFLMKREVRASVMIIFFAWLCSSVAVAPHIDIGLDQELSMPHDSFQLKYF 900
            ************************************************************
p50         QHLNRYLNIGPPVYFVLTDAETDAKLNYSTPDVQNLLCGSRFCRPDSLAMQIYAASRSPA 960
Nistari     QHLNRYLNIGPPVYFVLTDAETDAKLNYSTPDVQNLLCGSRFCRPDSLAMQIYAASRSPA 960
Cell        QHLNRYLNIGPPVYFVLTDAETDAKLNYSTPDVQNLLCGSRFCRPDSLAMQIYAASRSPA 960
C108        QHLNRYLNIGPPVYFVLTDAETDAKLNYSTPDVQNLLCGSRFCRPDSLAMQIYAASRSPA 960
JingSong    QHLNRYLNIGPPVYFVLTDAETDAKLNYSTPDVQNLLCGSRFCRPDSLAMQIYAASRSPA 960
            ************************************************************
p50         DTYIAAPANSWLDDFFDWSVSPECCFYFQSNNSFCPSDTQPDDCAACNIKLVQPEERPSP 1020
Nistari     DTYIAAPANSWLDDFFDWSVSPECCFYFQSNNSFCPSDTQPDDCAACNIKLVQPEERPSP 1020
Cell        DTYIAAPANSWLDDFFDWSVSPECCFYFQSNNSFCPSDTQPDDCAACNIKLVQPEERPSP 1020
C108        DTYIAAPANSWLDDFFDWSVSPECCFYFQSNNSFCPSDTQPDDCAACNIKLVQPEERPSP 1020
JingSong    DTYIAAPANSWLDDFFDWSVSPECCFYFQSNNSFCPSDTQPDDCAACNIKLVQPEERPSP 1020
            ************************************************************
p50         QDFSTYLPYFLQDNPSSRCVKGGHAAYSHAVNLRSHSQNTTGIGATYYQSYHTVLRSSSD 1080
Nistari     QDFSTYLPYFLQDNPSSRCVKGGHAAYSHAVNLRSHSQNTTGIGATYYQSYHTVLRSSSD 1080
Cell        QDFSTYLPYFLQDNPSSRCVKGGHAAYSHAVNLRSHSQNTTGIGATYYQSYHTVLRSSSD 1080
C108        QDFSTYLPYFLQDNPSSRCVKGGHAAYSHAVNLRSHNQNTTGIGATYYQSYHTVLRSSSD 1080
JingSong    QDFSTYLPYFLQDNPSSRCVKGGHAAYSHAVNLRSHNQNTTGIGATYYQSYHTVLRSSSD 1080
            ************************************.***********************
p50         YYGSLRAARALAGSLTETLNAHLRDLGHSATVNVFPYSVFYVFYEQYLTMWSDTLKSMGI 1140
Nistari     YYGSLRAARALAGSLTETLNAHLRDLGHSATVNVFPYSVFYVFYEQYLTMWSDTLKSMGI 1140
Cell        YYGSLRAARALAGSLTETLNAHLRDLGHSATVNVFPYSVFYVFYEQYLTMWSDTLKSMGI 1140
C108        YYGSLRAARALAGSLTETLNAHLRDLGHSATVNVFPYSVFYVFYEQYLTMWSDTLKSMGI 1140
JingSong    YYGSLRAARALAGSLTETLNAHLRDLGHSATVNVFPYSVFYVFYEQYLTMWSDTLKSMGI 1140
            ************************************************************
p50         SVLSIFFVTFVLMGFDLFSALVVVVTITMIVVNLGGLMYWWGISLNAVSLVNLVMAVGIS 1200
Nistari     SVLSIFFVTFVLMGFDLFSALVVVVTITMIVVNLGGLMYWWGISLNAVSLVNLVMAVGIS 1200
Cell        SVLSIFFVTFVLMGFDLFSALVVVVTITMIVVNLGGLMYWWGISLNAVSLVNLVMAVGIS 1200
C108        SVLSIFFVTFVLMGFDLFSALVVVVTITMIVVNLGGLMYWWGISLNAVSLVNLVMAVGIS 1200
JingSong    SVLSIFFVTFVLMGFDLFSALVVVVTITMIVVNLGGLMYWWGISLNAVSLVNLVMAVGIS 1200
            ************************************************************
p50         VEFCSHLVHSFSVSAGRGRGERAADALLRMGSSVLSGITLTKFGGIIVLATAKSQIFQVF 1260
Nistari     VEFCSHLVHSFSVSAGRGRGERAADALLRMGSSVLSGITLTKFGGIIVLATAKSQIFQVF 1260
Cell        VEFCSHLVHSFSVSAGRGRGERAADALLRMGSSVLSGITLTKFGGIIVLATAKSQIFQVF 1260
C108        VEFCSHLVHSFSVSAGRGRGERAADALLRMGSSVLSGITLTKFGGIIVLATAKSQIFQVF 1260
JingSong    VEFCSHLVHSFSVSAGRGRGERAADALLRMGSSVLSGITLTKFGGIIVLATAKSQIFQVF 1260
            ************************************************************
p50         YFRMYLGIVLFGAAHGLVFLPVMLSYIGSPVNKQKLANQRRRGNETTVAESSLTRVRHGH 1320
Nistari     YFRMYLGIVLFGAAHGLVFLPVMLSYIGSPVNKQKLANQRRRGNETTVAESSLTRVRHGH 1320
Cell        YFRMYLGIVLFGAAHGLVFLPVMLSYIGSPVNKQKLANQRRRGNETTVAESSLTRVRHGH 1320
C108        YFRMYLGIVLFGAAHGLVFLPVMLSYIGSPVNKQKLANQRRRGNETTVAESSLTRVRHGH 1320
JingSong    YFRMYLGIVLFGAAHGLVFLPVMLSYIGSPVNKQKLANQRRRGNETTVAESSLTRVRHGH 1320
```

```
               ************************************************************
p50            PTYYREYTFDSIPS*    1334
Nistari        PTYYREYTFDSIPS*    1334
Cell           PTYYREYTFDSIPS*    1334
C108           PTYYREYTFDSIPS*    1334
JingSong       PTYYREYTFDSIPS*    1334
               ***************
```
