# Supplementary figures and images for: Identification and Characterization of Genes Related to Resistance of Autographa californica Nucleopolyhedrovirus Infection in Bombyx mori
Source: Insects. 2022 May 6;13(5):435. doi: 10.3390/insects13050435 (PMC9144136; doi:10.3390/insects13050435)

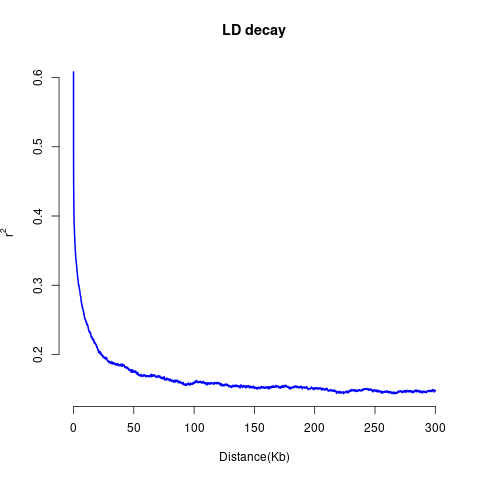

Supplement: Supplementary file 1 [file insects-13-00435-s001.zip › Figure S1.png]

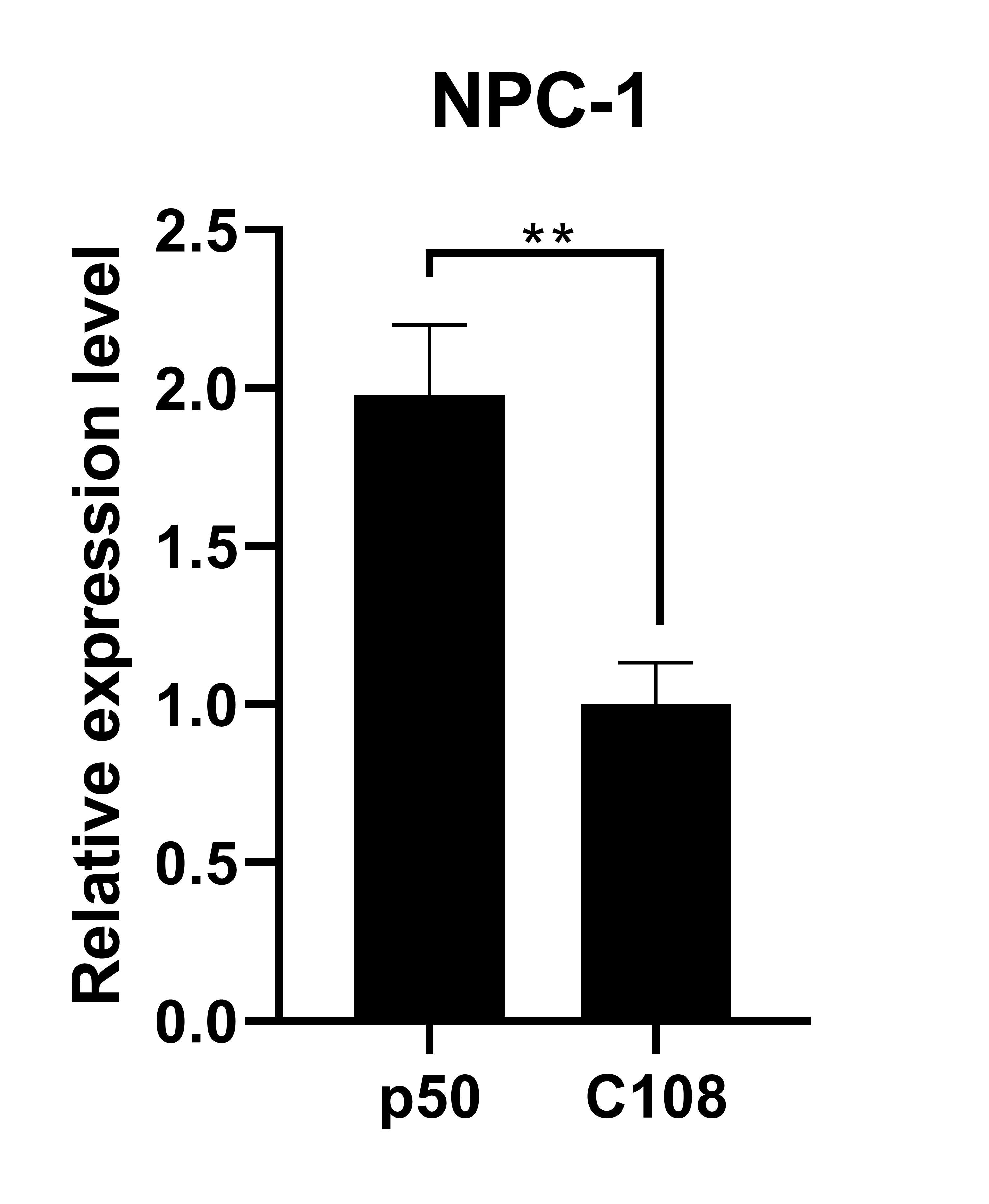

Supplement: Supplementary file 1 [file insects-13-00435-s001.zip › Figure S2.tif]
